# UAV-TIRVis: A Benchmark Dataset for Thermal–Visible Image Registration from Aerial Platforms

**DOI:** 10.3390/jimaging11120432

**Published:** 2025-12-04

**Authors:** Costin-Emanuel Vasile, Călin Bîră, Radu Hobincu

**Affiliations:** Department of Electronic Devices, Circuits and Architectures, Faculty of Electronics, Telecommunications and Information Technology, National University of Science and Technology Politehnica Bucharest, 060042 Bucharest, Romania; costin.vasile1003@upb.ro (C.-E.V.); calin.bira@upb.ro (C.B.)

**Keywords:** thermal–visible, image registration, UAV dataset, cross-spectral alignment, benchmark dataset, ORB, SIFT, SURF, KAZE

## Abstract

Registering UAV-based thermal and visible images is a challenging task due to differences in appearance across spectra and the lack of public benchmarks. To address this issue, we introduce UAV-TIRVis, a dataset consisting of 80 accurately and manually registered UAV-based thermal (640 × 512) and visible (4K) image pairs, captured across diverse environments. We benchmark our dataset using well-known registration methods, including feature-based (ORB, SURF, SIFT, KAZE), correlation-based, and intensity-based methods, as well as a custom, heuristic intensity-based method. We evaluate the performance of these methods using four metrics: RMSE, PSNR, SSIM, and NCC, averaged per scenario and across the entire dataset. The results show that conventional methods often fail to generalize across scenes, yielding <0.6 NCC on average, whereas the heuristic method shows that it is possible to achieve 0.77 SSIM and 0.82 NCC, highlighting the difficulty of cross-spectral UAV alignment and the need for further research to improve optimization in existing registration methods.

## 1. Introduction

Image registration is a critical preprocessing step in image analysis, defined as the process of geometrically aligning two or more images of the same scene acquired at different times, from various viewpoints, or by different sensors [1].

Differences in imaging conditions, such as sensor modality, acquisition geometry, or temporal variation, introduce spatial and radiometric inconsistencies that challenge direct comparison or fusion. Registration compensates for these discrepancies, thereby enabling integrated interpretation and analysis.

Image registration is essential across several domains. In remote sensing, the increasing availability of heterogeneous Earth observation data, particularly from optical and synthetic aperture radar (SAR) sensors, has highlighted the importance of robust registration techniques. While SAR imagery provides structural detail and weather-independent acquisition, optical imagery contributes spectral richness and visual interpretability. Their fusion, achievable only through accurate registration, supports advanced applications such as land cover classification, vegetation monitoring, and disaster response [2]. Last but not least, it could be applied to digital image stabilization [3] since image registration is geometrically related to it, via affine transforms.

In multimodal imaging, where data are acquired across different spectral bands or sensor types (e.g., visible–infrared, RGB–LiDAR), registration ensures that complementary information is mapped into a common coordinate system. This alignment is indispensable for tasks such as environmental monitoring, urban inspection, surveillance, and autonomous navigation [4]. Similarly, in multi-temporal studies, registration facilitates the detection of subtle changes over time, enabling applications ranging from monitoring tumor evolution in medical imaging to assessing urban expansion and landscape dynamics in remote sensing.

The main goal of image registration is to determine the transformations to be applied to the moving (source) image so that it aligns as closely as possible with the reference (target) image. These transformations must compensate for differences in viewpoint, scale, rotation, warping, and any other geometric or radiometric discrepancies between the images.

Systematic optical distortions (e.g., barrel, chromatic aberration, vignetting) are typically corrected beforehand, while registration algorithms focus on geometric misalignments and scene-dependent deformations.

In practice, image registration typically follows a sequence of well-defined steps:

- Preprocessing (often in multimodal, since the images come from different sensors: SAR vs. optical, IR vs. visible, CT vs. MRI)
- Keypoint detection (as invariant as possible: points (e.g., corners), edges (e.g., contours), regions (e.g., anatomical structures) in both images
- Keypoint matching
- Transform estimation
- Resampling and Image Warping (apply the transformation to align the source with the target)
- Evaluation of Registration Accuracy (if ground truth exists)

To achieve alignment, several methodological paradigms have been developed, ranging from traditional optimization-based techniques to modern learning-driven approaches:

- Area-based: the alignment is found by optimizing a similarity measure (cross correlation, mean squared error, mutual information, structural similarity index, etc.)
- Feature-based: identify distinctive points, lines, or regions in both images and match them to estimate the transformation
- Intensity-based: directly compare pixel intensities across images using similarity metrics like normalized cross-correlation, mutual information, or sum of squared differences.
- Learning-based: use Neural Networks to predict deformation fields
- Hybrid: combine at least two of the above

## 2. Related Work

A variety of medical datasets have been introduced to benchmark image registration across organs and modalities. These include large-scale resources such as Learn2Reg for multi-task challenges, COPDgene and SegTHOR for thoracic CT, and multimodal abdominal collections (Duke CVIT [5]). Specialized ophthalmic datasets like FIRE, FLoRI21, COph100, and MEMO target retinal registration, while some extend to histopathology and broader multimodal biomedical settings.

The only publicly available datasets for UAV-originated image registration are H2OPM [6] and MTV [7]. The H2OPM Image Registration Dataset is a dataset for evaluating registration methods. It consists of 8 reference orthophoto maps (OPMs) of urban and non-urban areas in Austria. For each reference image, 3–11 historical aerial images captured between May 1943 and May 1945 are available, yielding a total of 42 image pairs. To evaluate registration accuracy, manually selected ground-truth correspondences between historical images and the OPM are provided [6]. A more recent UAV-based dataset, the Multi-View Thermal–Visible Image Dataset by Liu et al. [7], introduces over 40k cross-spectral image pairs registered via a semi-automatic 3D reconstruction pipeline. Thermal images are manually aligned to selected visible views, and the resulting transformations are propagated through multi-view geometry to obtain dense supervision and 6-DoF camera poses. This dataset is particularly suited for training learning-based cross-spectral matching or pose estimation networks. In contrast, UAV-TIRVis provides manually verified 2D ground-truth registrations for every image pair, enabling direct evaluation of registration algorithms in the image domain. Therefore, UAV-TIRVis complements large-scale pose-supervised datasets by emphasizing pixel-level geometric accuracy and reproducible manual annotation.

The development of robust multimodal UAV image registration methods has been hindered by the lack of publicly available datasets that pair thermal and visible imagery under real-world conditions. Existing resources primarily focus on medical registration tasks, leaving a critical gap in cross-spectral UAV imaging that we address in this paper. Table 1 highlights the existing public image registration datasets and compares them based on the modality, year, size, and ground truth availability.

Our work addresses the literature gap by proposing a benchmark dataset for multimodal UAV registration. We offer synchronized, annotated, and challenging image pairs that enable fair evaluation, stimulate methodological advances, and foster reproducibility in a rapidly growing research domain.

We also investigated the potential use cases of our dataset. Table 2 provides a comparative overview of recent image registration methods relevant to both remote sensing and multimodal UAV applications. The selected works span classical feature-based approaches, LiDAR–camera calibration pipelines, and modern learning-based registration frameworks. They cover diverse sensing modalities (visible, thermal, infrared, LiDAR) and application domains (UAV mapping, robotic perception, medical and industrial imaging). The table highlights whether the methods are open-source, their core technical characteristics, and provides practical remarks about computational requirements, flexibility, and domain transferability.

From this comparison, several trends emerge. First, learning-based methods such as LoFTR, VoxelMorph, and DeepReg have demonstrated state-of-the-art accuracy and adaptability, especially for dense or non-rigid registration. Still, they often remain domain-specific (e.g., medical imaging). Among them, LoFTR stands out as the only widely open-source transformer-based model suitable for adapting to UAV thermal–visible registration. Second, classical feature-based approaches—including SIFT variants, ORB-based pipelines, and hybrid detectors—remain attractive for lightweight UAV implementations because of their efficiency, interpretability, and independence from large training datasets. Traditional methods, such as SIFT and ORB-based registration, demonstrate that carefully designed descriptors can still achieve subpixel or a few-pixel accuracy at a low computational cost. Third, domain-specific adaptations demonstrate that combining coarse-to-fine strategies with modality-invariant descriptors yields practical performance in cross-modal environments. Finally, LiDAR–camera calibration frameworks such as SE-Calib and NRLI-UAV point toward the growing importance of multisensor fusion and the potential transfer of these techniques to thermal–visible UAV systems.

Overall, the landscape reflects a clear trade-off between accuracy and deployability: deep learning frameworks offer precision and flexibility at the cost of resources and data, while traditional feature-based or hybrid methods provide practical, fast, and robust solutions for embedded UAV or field applications.

## 3. Methods

This section describes the methods used to acquire the dataset, perform manual registration, and evaluate automated image registration techniques.

### 3.1. Dataset Acquisition

The images were captured using a DJI Mavic 3T drone equipped with both a visible-spectrum camera and a thermal camera. Visible-spectrum images are captured at 4000 × 3000, while thermal images are captured at 640 × 512. To examine how image size impacts the registration process, a small subset of the thermal images was saved in a 1280 × 1024 format. The camera orientation was set to nadir (pointing straight down), while horizontal speed and altitude varied depending on location, scenario, and the targeted objects.

**Figure 1:**
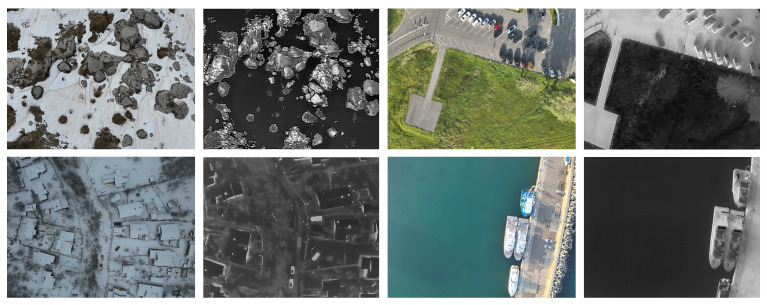
Example image pairs (visible and thermal) contained in our dataset.

The dataset comprises 80 visible–thermal image pairs captured in challenging scenarios for automatic image registration. The scenarios include residential areas with high construction density (houses), mountainous areas, and the seaside. Figure 1 displays four pairs of images, each consisting of a visible spectrum image and a thermal image, that illustrate these challenging scenarios. Each case from our public dataset contains:

- 4000 × 3000 visible-spectrum image
- 640 × 512 thermal image
- 4000 × 3000 warped thermal image
- 4000 × 3000 overlay of the warped thermal over the visible image
- The coordinates of the keypoints used to register the images

### 3.2. Manual Image Registration

**Figure 2:**
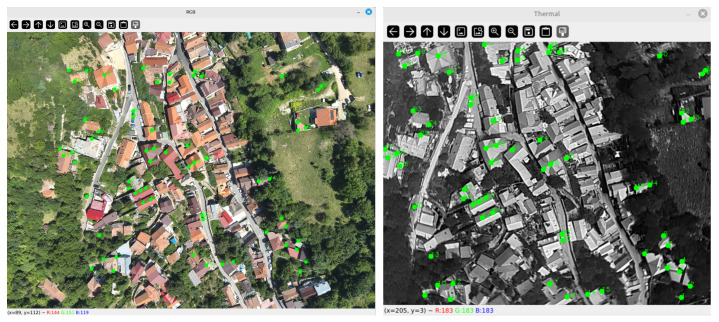
Landmark matching example with 65 pairs of points (marked with green). The numbers in the pictures depict the order the pairs were matched manually (small numbers first).

Each visible–thermal pair is manually aligned using an interactive GUI developed in Python 3.11. The visible image (reference) and the thermal image (moving) are displayed side by side, and the operator selects a set of corresponding landmarks that can be visually identified in both modalities (e.g., building corners, road intersections, edges of roofs, or other structurally consistent features). Typically, 40–80 landmarks are placed per pair. This process is illustrated in Figure 2.

After each selection, the framework overlays the current warped thermal image on the visible image, allowing the user to assess the residual misalignment. Given the paired landmarks, the framework estimates a smooth non-rigid transformation using thin-plate spline interpolation. This transformation accounts for local geometric differences between the thermal and visible spectrum images, which may arise from variations in sensor placement, field of view, or lens characteristics. Unlike rigid or projective models, the non-rigid formulation allows correction of local misalignments while maintaining global smoothness. This was achieved using the radial basis function (Rbf) implementation provided in SciPy’s interpolation module [28].

**Figure 3:**
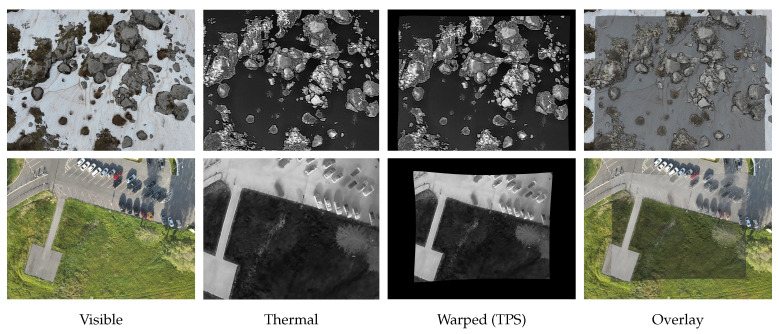
Thermal–visible registration results for two scenes.

Once the transformation is computed, the thermal image is warped into the coordinate space of the visible spectrum image. The aligned thermal data can then be directly compared with, or fused with, the visible-spectrum image. For qualitative assessment, the framework also generates an overlay that blends thermal intensities with the visual scene, enabling a straightforward visual evaluation of registration accuracy. Figure 3 presents two examples of the input images along with the resulting warped and overlaid images.

**Figure 4:**
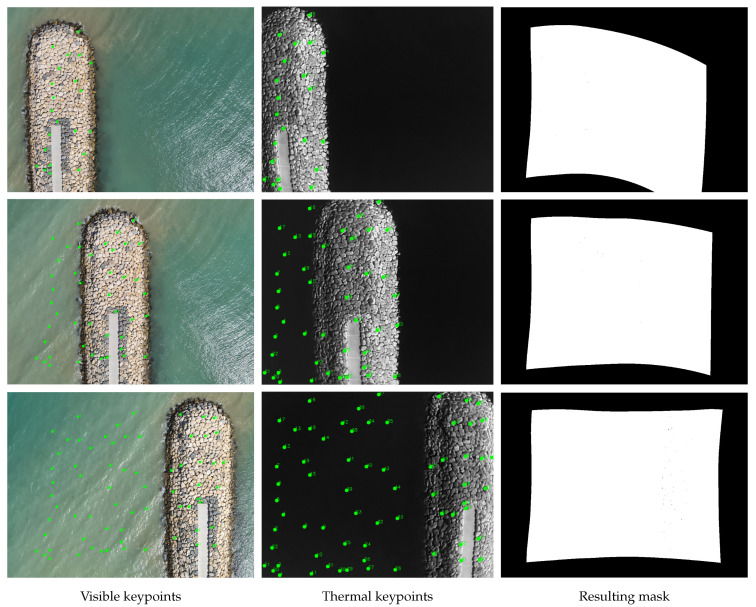
Example of iterative manual landmark matching. Each row represents a different iteration. The numbers in the figures indicate the order in which the pairs were manually matched (small numbers first).

Finally, the framework supports the storage and reuse of landmark sets, ensuring that registration results are reproducible and can be refined iteratively. This is also useful for image pairs acquired under similar conditions by using the final landmark set of one pair as a reference point for another pair. Figure 4 shows an example where multiple iterations were required to create an accurate mask.

The process of manual registration is summarized below:

1. Display the visible (reference) and thermal (moving) images side by side in an interactive GUI
2. Manually select corresponding landmarks on both images (typically 40–80 per pair, but could go up to 100 or more depending on the scenario complexity), using easily identifiable structures such as building corners or roads
3. Estimate a smooth non-rigid transformation using a thin-plate spline (TPS) based on the selected landmark pairs
4. Warp the thermal image according to the TPS transformation to align it with the visible image
5. Inspect the overlay; if misalignment remains, add or adjust landmarks and recompute the TPS (iterative refinement)
6. Once alignment is satisfactory, save the landmark coordinates and TPS parameters for reproducibility
7. Generate the final warped thermal image (4000 × 3000) and a blended overlay

### 3.3. Annotation Accuracy and Consistency

To assess the robustness of the manual registration process, we conducted three experiments: a leave-one-out experiment was performed to evaluate landmark influence, a perturbation analysis for transformation stability, and an inter-annotator consistency measurement. The results are presented below.

- Landmark influence (LOO). A leave-one-out experiment was performed, in which each landmark was removed in turn and the thin-plate spline (TPS) transformation was recomputed. The resulting warped image differed from the full-set warp by an average of 25.48 px on the 4000 × 3000 visible grid, confirming that each selected landmark contributes substantially to the global alignment.
- Transformation stability. Introducing small random deviations to landmark positions yielded 95% confidence intervals of 3.48 px (x) and 3.28 px (y), demonstrating that the estimated TPS transformation is highly stable to minor landmark uncertainty.
- Inter-annotator consistency. Although the published dataset was annotated by a single experienced annotator, expanding it to a significantly larger scale would require the involvement of multiple annotators. To assess this, we evaluated inter-annotator accuracy by comparing the points selected by two additional annotators with those provided by the trained annotator. The mean landmark discrepancy between annotators was 4.49 px for visible images and 0.84 px for thermal images, demonstrating strong consistency among operators and confirming the high reproducibility of the manual registration framework, even when used by newly trained annotators.

### 3.4. Automated Image Registration

To highlight the significance of our dataset and demonstrate its intended usage, we evaluated several automated image registration algorithms using it. We evaluated four feature-based methods: ORB, SURF, SIFT, and KAZE. Additionally, we examined a cross-correlation method, an intensity-based registration method, and a custom heuristic intensity-based correlation method. This evaluation was conducted on a subset of the master dataset, which includes a total of 41 images. The breakdown of the images is as follows: 7 images from a mountain scenario, 14 images featuring a seaside pier, 6 images from a residential area, and 14 images depicting a mountain resort. Our repository contains all the information used in this process, including the dataset subset, MATLAB 2025a scripts for each registration method, and the resulting images and statistics.

The feature-based methods (ORB, SURF, SIFT, and KAZE) were applied by extracting the associated keypoints using the MATLAB built-in functions (*detectORBFeatures*, *detectSURFFeatures*, *detectSIFTFeatures*, *detectKAZEFeatures*). The resulting keypoints were passed through a pipeline of MATLAB built-in functions for keypoints filtering (*selectStrongest*), feature extraction (*extractFeatures*), feature matching (*matchFeatures*), geometric transform estimation (*estimateGeometricTransform2D*), and finally image warping (*imwarp*).

The cross-correlation and intensity-based methods were applied by estimating the 2D transformation using the built-in MATLAB functions *imregcorr* (cross-correlation) and *imregtform* (intensity-based), and then warping the thermal images accordingly.

It is essential to note that the purpose of these experiments is to demonstrate the behavior of existing registration techniques on the UAV-TIRVis dataset, highlighting its intended use case and how traditional methods perform in such scenarios. To ensure that the dataset is neither trivial nor inconsistent, we also included a simple heuristic “enhanced intensity-based” approach that empirically explores different image scaling factors before applying standard registration. This method is not theoretically motivated but serves as a practical demonstration that acceptable registration accuracy can be achieved. This method is described in the following paragraph.

The heuristic intensity-based method is based on the MATLAB *imregister* function. In our initial experiments, we observed that resizing the thermal image by different factors could improve registration quality. To explore this further, we implemented a loop that performs registration after resizing the thermal image using factors ranging from 1 to 6, with increments of 1. After each iteration, the metrics listed in the following subsection are calculated, and the best registration result is stored. This method can be summarized as follows:

- For each scale factor f in trialFactors:
  – Resize moving image by *f*
  – Estimate similarity transform (moving → ref)
  – Refine with affine registration (moving → ref)
  – Obtain warped moving image
  – Compute metrics: RMSE, PSNR, SSIM, NCC
  – If metrics are better than bestResult (by NCC, tie-break SSIM), update stored factor, warped image, and metrics as bestResult
- End for
- Save the best warped image and overlays
- Save timing logs

Preprocessing. All methods involved two preprocessing steps: first, converting the images to grayscale with the *im2gray* function, and second, converting them to single-precision with the *im2single* function.

To enhance registration accuracy while minimizing computational cost, we perform a meet-in-the-middle resizing step, in which both images are scaled so that their longest dimension matches the average of the two, up to a maximum of 1600 pixels, while preserving the original aspect ratio.

The custom heuristic approach included a resizing step, with factors ranging from 1 to 6.

### 3.5. Metrics

The quality of the results obtained using the automated image registration techniques described in the previous subsection was assessed using four metrics: Root Mean Squared Error (RMSE), Peak Signal-to-Noise Ratio (PSNR), Structural Similarity Index Measure (SSIM), and Normalized Cross Correlation (NCC).

**Figure 5:**
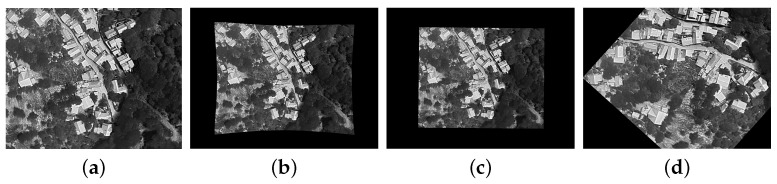
Example of images used to compute the metrics. (**a**) source image captured by the UAV; (**b**) manually warped image used as ground truth; (**c**,**d**) examples of images resulting from the automated registration process. The metrics are computed by comparing the images warped using the automated process (**c**,**d**) with the ground truth (**b**). All warped images (**b**–**d**) are generated from (**a**); therefore, they share the same modality and can be safely compared without additional conversions.

These metrics were computed by comparing the warped images from the automated registration process with the warped thermal images from manual image registration, which were considered ground truth Figure 5.

For each image, these metrics were computed, then averaged across the images in each scenario and across the entire dataset. This would highlight the scenarios in which existing automated registration methods yield acceptable results and those in which further improvements are still required to achieve better registration quality.

In addition to the quantitative measures, a subjective evaluation (referred to as Subjective Acceptability) was also conducted to assess the perceived quality of the registrations. For each automatically registered image, we visually examined the alignment between the visible and thermal modalities and categorized the result as either acceptable or unacceptable. The number of images deemed acceptable was then counted and expressed as a proportion of the total number of tested cases, for each registration method and scenario. This qualitative assessment complements the objective metrics by providing an intuitive measure of practical usability and visual plausibility of the registration results.

## 4. Evaluation

**Figure 6:**
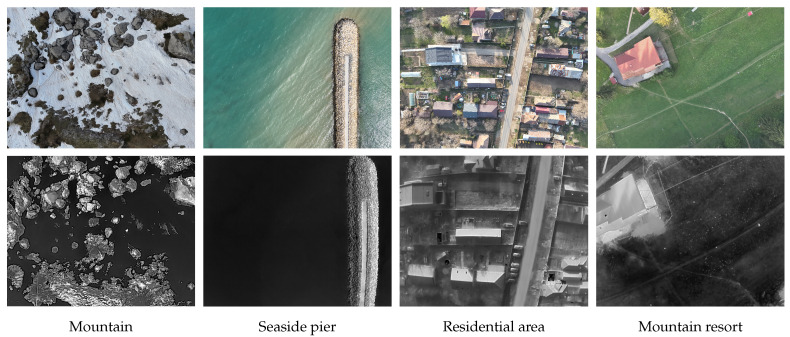
Representative visible–thermal pairs for the four scenarios used in the evaluation process. **First row**: visible images; **second row**: the associated thermal images.

This section presents the results we obtained from evaluating different registration methods on our dataset. It emphasizes the importance of such datasets and the need for increased efforts in the field of automated image registration for UAV scenery. For this evaluation, we selected a subset of our dataset, containing multiple images acquired in the following scenarios: mountain, seaside pier, residential areas, and mountain resort. Figure 6 displays four pairs of images, with each pair representing a different scenario used in the evaluation process.

These scenarios were selected based on our expectation that they would present distinct challenges for the registration process. The first type involves a high density of objects, such as in a residential area, where complex 2D transformations are necessary for precise warping. The second type consists of areas with low information, like piers, rocky mountains, and snowy landscapes, which have a limited number of keypoints that can be automatically detected and utilized for estimating 2D transformations.

### 4.1. Traditional Registration Methods

Table 3 reports metrics averaged over the entire dataset. The results indicate that conventional registration approaches perform poorly, yielding less than 0.6 NCC between the automated outputs and the ground-truth images produced by the manual registration pipeline described in the previous section. Although some methods perform acceptably in specific scenarios (demonstrated by the results presented below), these results show there is no one-size-fits-all solution for the specialized task of UAV registration of 640p thermal imagery onto 4K visible frames using traditional and straightforward approaches. Table 3 indicates that the heuristic method achieved the highest performance results, with a normalized cross-correlation (NCC) exceeding 0.8 and a structural similarity index (SSIM) of 0.77. This demonstrates that achieving acceptable registration accuracy is possible in these scenarios, but not with conventional approaches.

Table 4 presents the metrics averaged across scenarios, illustrating the performance of each registration method in every case. This analysis demonstrates that although traditional registration methods cannot generalize across scenarios, some can still yield acceptable results in specific situations. The SURF, KAZE, intensity-based, and correlation-based methods achieved an NCC of approximately 0.7 or more in one scenario. However, ORB and SIFT did not produce acceptable results in any case. The heuristic produced the best results in every scenario, demonstrating its robustness across various environments.

The subjective acceptability (SAC) correlates strongly with the NCC value, indicating that the NCC is an effective metric for assessing registration accuracy.

To ensure a fair comparison of the different registration methods, we also evaluated the duration of each method. The results are presented in Table 5. While our method has demonstrated superior results across all scenarios in the dataset, it comes with a significant drawback: its execution time is substantially slower than that of the other evaluated methods. This suggests that a greater research effort is needed to develop more effective algorithms suitable for these applications, highlighting a potential gap in the existing literature.

The analysis of the obtained results revealed that the performance of individual metrics was not consistent across all scenarios. Specific metrics yielded high values in some scenes but performed poorly in others, indicating that their reliability depends on image characteristics such as texture richness, contrast, and alignment with modality. In some cases, even images belonging to the same scenario or to visually similar sets exhibited noticeable variations in metric values. This suggests that no single metric can universally reflect registration quality across all conditions.

### 4.2. Other Automated Registration Methods

In addition to the traditional registration methods mentioned above, we also evaluated several alternative methods. These were excluded from the previous results due to their poor performance; however, we believe it is important to mention them as a starting point for future research. This section provides a brief overview of some of these methods and their associated results.

Cross-modal registration algorithms, such as RIFT [29] and CFOG [30], are algorithms designed for visible–infrared image matching. On our dataset, RIFT achieved very low similarity scores (average NCC < 0.01), and CFOG proved unsuitable due to the large-scale mismatch between the thermal (640 × 512) and visible (4000 × 3000) images. CFOG assumes comparable spatial resolution and fixed feature grid sizes, which are not directly compatible with the multi-scale UAV setup. These observations further underline the distinct challenges posed by UAV-based cross-spectral imagery, where differences in perspective, scale, and spectral response exceed those in most existing datasets. Due to the inadequate performance of RIFT and CFOG on this complex task, we have chosen not to include them in the results presented in Section 4.

Learning-based methods rely on supervised training using dense correspondence ground truth across modalities. Since UAV-TIRVis introduces these types of data for the first time in aerial cross-spectral registration, no pretrained models currently exist for this domain. Training new ones would require hundreds of paired samples, making such exploration more appropriate as future research built upon this dataset.

**Figure 7:**
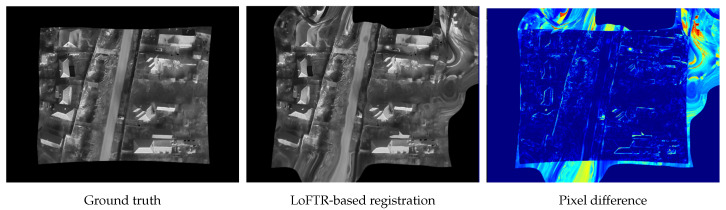
Visual representation of the LoFTR-based registration results. The pixel-difference image uses a color scale in which cold colors (blue) indicates identical pixels, and warmer colors indicate increasing levels of difference (e.g., red indicates the largest pixel discrepancy).

Although there are many learning-based methods in the literature [19,22,23], the official LoFTR [20] repository contains a pretrained model that can be used to assess the performance of this method. Figure 7 shows a visual representation of the results we achieved using this method. Considering that no fine-tuning was performed, these results indicate that these methods hold significant potential for such applications and suggest that future research in this area is encouraged. This is further supported by our analysis of the same dataset subset, which is highlighted in the Table 6.

## 5. Discussion

This section aims to highlight and discuss the complexity of the heuristic approach introduced above, investigating whether the acceleration of this algorithm could enable achieving reasonable registration speeds, transforming it from an exploratory method to a practical one.

Although discovered experimentally, the “resize” step in the heuristic approach makes sense as image registration is a hard optimization task: scaling would correct image scale and field-of-view; down-scaling would smooth noise and simplify the optimization landscape (which is a big problem when cross-modal registration is performed: heat-image does not have the same rough edges/corners as visible-spectrum image); up-scaling via interpolation might help by providing continuous gradients, and provide consistent evaluation as the metrics are computed on high-res reference.

Per-step time complexity of this method (where N is the number of pixels) is listed below:

- Resize moving image by *f*: $O(k_{f}\;N)$
- Estimate similarity transform (moving → ref, imregtform): $O(k_{\mathrm{sim}}\;N)$
- Refine with affine registration (moving → ref, imregister): $O(k_{\mathrm{aff}}\;N)$
- Obtain warped moving image (final resampling onto ref grid): O(N)
- Compute metrics (RMSE, PSNR, SSIM, NCC) on aligned images: O(N)
- Compare/update bestResult (by SSIM, tie-break NCC): O(1)

Regarding the acceleration of the heuristic algorithm containing a hybrid MIMD-SIMD code, by moving it to a GPU (or a massive parallel processor) instead of relying on a CPU [31], there are three main factors with a high impact: the number of independent cores (i.e., the number of different pixel kernels that can be run simultaneously), memory bandwidth, and compute power.

Our test computer, in addition to common components, features a CPU Ryzen 5 7600 from AMD (Santa Clara, CA, USA) with dual-channel DDR running at 4800 MT/s and an GPU GeForce 4700 Ti Super from Nvidia (Santa Clara, CA, USA). Since the CPU’s L3 cache is too small to contain all the data, RAM memory speed will limit CPU data transfers, whereas the GPU can contain in its local memory, all the input/output image data (such that transfers via PCIe will only occur at the very beginning and at the very end of the computation). A comparison of acceleration-relevant specifications between our CPU and GPU is shown in Table 7.

The independent cores specification is an indication of how many loop iterations one can perform in parallel (the loops differ in what they carry out at the same time, since the amount of data differ). The max compute power indicates the pixel processing power. That being said, since most operations are not more time-complex than O(N), it is our opinion that memory bandwidth is expected to impact the most the amount of acceleration we can obtain by porting our algorithm from CPU to GPU; thus, we do not expect more than 6.5× acceleration on a system comparable to ours.

An avenue to be investigated when software tools will be more mature, regards the quality trade-off, for switching from FP32 to FP16 computations: since the processing is mostly performed in O(N), a smaller data representation would imply less data transfers, therefore less computation time: this optimization would both apply to CPU (AVX512 extensions has some vectorized FP16 operations) and GPU (with appropriate tensor-compute units). While the tensor-core FP16 performance looks extremely compelling (for Nvidia Geforce RTX5090, 8x versus FP32), a word of caution: very few software tools/frameworks support the NVIDIA tensor-compute units (for example, OpenCV has barely adequate support for GPUs, via GpuMat, while FP16 support is only found in upload/download/format-conversion targeting DNN computations) and without them, GPU FP16 performance is similar to FP32, as the operations will be carried out using the CUDA cores; another remark would be that top tensor-core performance is stated for sparse 2:1 matrix calculations while dense matrix performance (which is usually the case for images), for the newest 5000-series, is only half that). Our current reasonably cost recommendation is to obtain the lowest-latency, highest-speed minimum of 4-channel DDR5 memory for CPU (min 256-bit total bus width), and the fastest and widest GPU memory (GDDR7 with 512-bit as in RTX5090), by sheer bandwidth, as this will increase performance at least three times as opposed to our system in both CPU-only or GPU-only scenarios.

Another take on memory bandwidth would be to use a server-class CPU (Xeon-family from Intel (SantaClara, CA, USA) or EPYC-family from AMD), with 256+ MB of L3 cache, which may transfer data at 3 TB/s, besting the Nvidia Geforce RTX3090’s larger local memory. For images reasonably small (12 MP grayscale image represented in FP16 takes up 24 MBytes), they might just fit the L3 data cache, with their intermediate representations, avoiding main RAM transfers at “only” 100 GB/s to 500 GB/s (for 12-channel DDR5). If the resolutions are higher, one may use the newer HBM-fitted processors, which, with more than 1 GB per core, can achieve even more than 1.5 TB/s transfer speeds. Both Intel and AMD are expected to launch processors with a high amount of HBM memory in the near future (and similarly higher-speed integrated high-bandwidth memory).

## 6. Conclusions

In this work, we introduce UAV-TIRVis, a benchmark dataset for thermal–visible image registration from aerial platforms, accompanied by a reproducible manual registration pipeline and a set of baseline automated registration experiments. We also included a heuristic-enhanced intensity-based method to demonstrate that the limitations observed in existing algorithms stem from the algorithms themselves rather than from any shortcomings of the dataset. The results show that conventional approaches struggle to generalize across scenes, often yielding sub-optimal cross-spectral alignment. At the same time, our enhanced intensity-based pipeline achieves higher accuracy at the cost of substantially greater runtime. These findings highlight both the difficulty of cross-spectral UAV registration and the need for methods that are simultaneously robust and computationally efficient. UAV-TIRVis aims to catalyze research in UAV-based cross-spectral image registration. Extending the dataset with additional locations, altitudes, seasons, and sensor payloads (e.g., LiDAR and SAR proxies) will further strengthen benchmarking. Future work will also include adapting and retraining learned cross-spectral matchers in UAV-TIRVis, which could help bridge the modality gap once sufficient training data are available.

## Figures and Tables

**Table 1:** Comparison of public image registration datasets.

Dataset	Domain/Modality	Year	Size Details	Ground Truth Notes
Learn2Reg [8]	Multi-task medical (CT, MR, US, histology)	2021–2022	Multiple datasets across anatomies	Labels + evaluation framework; challenge benchmark
COPDgene [9]	Thoracic CT (inhale/exhale)	2013	10 BH-CT pairs, ∼7k landmarks	Manually validated landmark pairs; deformable registration reference
SegTHOR [10]	Thoracic CT (organs at risk)	2020	60 3D CT scans (40 train, 20 test)	Manual segmentation of heart, aorta, trachea, esophagus
FIRE [11]	Retinal fundus images	2017	134 pairs from 129 images	Landmark correspondences; standard fundus benchmark
FLoRI21 [12]	Retinal fluorescein angiography	2021	15 reference-target pairs	Ground-truth alignments; longitudinal retina
COph100 [13]	Infant retinal fundus	2025	491 image pairs (100 eyes, multi-session)	Correspondences + vessel masks; infant disease progression
MEMO [14]	Retinal EMA + OCTA	2023	EMA/OCTA image pairs	Landmarks + segmentation; vessel-density mismatch
ATOM [15]	Histology-Organ mapping	2024	Histology subregions in 3D organ	Spatial localization of histo sections within organ context
Multimodal Abdomen [5]	Synthetic MRI, CT, CBCT	2020	CycleGAN-generated volumes	Perfect co-registration; validation dataset
H2OPM [6]	Aerial orthophoto maps (Austria)	2018	8 references, 42 historical pairs	Manual correspondences; groupwise aerial registration
MTV [7]	Multi-view thermal–visible images	2022	40k image pairs, 640 × 512	camera metadata, 3D reference model, depth map of the visible images, and 6-DoF pose of all images

**Table 2:** Comparison of recent methods related to image registration.

Source	Type	Open-Source	Comments/Remarks
[16] NRLI-UAV: Non-rigid registration of sequential raw laser scans and images for low-cost UAV LiDAR point cloud quality improvement	LiDAR + imagery registration	No	A two-step “coarse-to-fine” non-rigid registration method addressing the low precision of low-cost UAV LiDAR systems; Final registration error <1 pixel in image space and <0.13 m in object space; Complex setup
[17] SE-Calib: Semantic Edges based LiDAR–Camera Boresight Online Calibration in Urban Scenes	LiDAR–Camera Boresight Calibration	No	This LiDAR–Camera calibration pipeline can be extended to thermal–visible sensors (especially semantic feature extraction, target-free operation, and multi-frame optimization)
[18] Mobile-Seed: Joint Semantic Segmentation and Boundary Detection for Mobile Robots	Lightweight semantic segmentation	No	Since this is a lightweight semantic segmentation method, it could be adapted/quantized to run onboard (on the drone or on-field on an embedded device) and achieve close to real-time performance
[19] U-Net vs. Transformer: Is U-Net Outdated in Medical Image Registration?	Learning-based image registration	No	Claims that U-Net with sufficient receptive field might still perform very well on most image registration tasks
[20] LoFTR: Detector-Free Local Feature Matching with Transformers	Learning-based image registration	Yes	Strong empirical results; Can be heavy in terms of GPU memory and compute, especially for large images or high resolution
[21] Transformer-Based Local Feature Matching for Multimodal Image Registration	Learning-based image registration	No	Built on top of LoFTR; Cross-modality, cross-dimensional registration
[22] DeepReg: a deep learning toolkit for medical image registration	Learning-based image registration	Yes	Open-source toolkit written in Python 3 (TensorFlow 2-based) designed for deep-learning-based image registration, originally in the medical imaging domain. Does not offer pretrained models
[23] VoxelMorph: A Learning Framework for Deformable Medical Image Registration	Learning-based image registration	Yes	CNN-based learning framework for deformable (dense) image registration in the medical-imaging domain; Claimed to have fast inference once trained
[24] Multi-Modal Remote Sensing Image Registration Method Combining Scale-Invariant Feature Transform with Co-Occurrence Filter and Histogram of Oriented Gradients Features	Multi-modal SIFT-based registration	No	Novel modification of SIFT to suppresses texture variations while preserving structural information
[25] Fast Automatic Registration of UAV Images via Bidirectional Matching	Visible-Visible ORB-based registration	No	Built on top of the lightweight feature-matching algorithm ORB; Targeted to visible spectrum UAV scenery
[26] A Two-Stage Registration Strategy for Thermal–Visible Images in Substations	Thermal–Visible registration	No	Domain-specific focus (electrical substations); Claims sub-5 pixel error across 30 images
[27] A Hybrid Approach for Image Acquisition Methods Based on Feature-Based Image Registration	Visible-Visible registration	No	Novel hybrid feature-detection/registration method aimed at image acquisition scenarios; Claimed to yield improved keypoint detection and computational efficiency compared to the conventional detectors (SIFT, ORB, etc.)

**Table 3:** Averaged results across the entire dataset for each registration method.

Method	RMSE	PSNR (dB)	SSIM	NCC
ORB	0.25	12.31	0.46	0.26
SURF	0.23	13.51	0.52	0.38
SIFT	0.19	15.38	0.60	0.53
KAZE	0.19	14.78	0.54	0.50
Cross-correlation	0.20	15.10	0.55	0.51
Intensity-based	0.22	13.94	0.53	0.29
Heuristic method *	0.12	18.53	0.77	0.82

* Our proposed method.

**Table 4:** Averaged results for four challenging scenarios.

Location	Method	RMSE	PSNR (dB)	SSIM	NCC	SAC **
	ORB	0.33	9.67	0.25	0.09	0/7
	SURF	0.28	11.07	0.33	0.13	1/7
	SIFT	0.30	10.42	0.25	0.08	0/7
Mountain	KAZE	0.30	10.36	0.27	0.05	0/7
	Cross-correlation	0.29	10.84	0.39	0.11	2/7
	Intensity-based	0.15	16.48	0.66	0.69	5/7
	Heuristic method *	0.13	17.52	0.72	0.79	7/7
	ORB	0.21	14.07	0.50	0.38	2/14
	SURF	0.23	13.12	0.46	0.29	1/14
	SIFT	0.14	18.13	0.70	0.68	10/14
Seaside pier	KAZE	0.16	15.86	0.50	0.56	2/14
	Cross-correlation	0.12	18.95	0.66	0.71	9/14
	Intensity-based	0.19	14.45	0.49	0.29	3/14
	Heuristic method *	0.11	18.88	0.78	0.78	11/14
	ORB	0.21	13.57	0.61	0.40	3/6
	SURF	0.13	17.58	0.79	0.81	5/6
	SIFT	0.15	16.61	0.74	0.72	5/6
Residential area	KAZE	0.12	17.92	0.80	0.83	6/6
	Cross-correlation	0.19	15.9	0.67	0.64	4/6
	Intensity-based	0.3	10.49	0.43	−0.09	0/6
	Heuristic method *	0.11	18.92	0.81	0.86	6/6
	ORB	0.27	11.59	0.48	0.20	3/14
	SURF	0.23	13.37	0.57	0.42	5/14
	SIFT	0.20	14.56	0.61	0.54	6/14
Mountain resort	KAZE	0.19	14.57	0.61	0.54	6/14
	Cross-correlation	0.24	13.03	0.48	0.44	3/14
	Intensity-based	0.23	13.64	0.54	0.25	4/14
	Heuristic method *	0.11	19.51	0.79	0.88	14/14

* Our proposed method. ** Subjective Acceptability: number of images judged as acceptably registered.

**Table 5:** Execution duration for each registration method.

Method	ORB	SURF	SIFT	KAZE	Cross-Corr	Intensity	Ours
Duration (s)	1.50	1.76	1.77	2.64	3.21	2.25	92.72

**Table 6:** Evaluation results using the LoFTR method.

Location	NCC	SAC **
Mountain	0.37	2/7
Seaside pier	0.70	11/14
Residential area	0.60	5/6
Mountain resort	0.54	8/14

** Subjective Acceptability: number of images judged as acceptably registered.

**Table 7:** CPU/GPU/RAM specifications of our test system.

Specification	CPU	GPU	Comments
Independent Cores	6	60	SIMD-vectorization on CPU (16× fp32) is inferior to CUDA cores per SM (128×)
Memory bandwidth (GB/s)	76.8	504.2	dual-channel, DDR 4800 MT/s 64b per Transfer = 76.8 GB/s
Max compute power (TFlops, 32-bit)	1	40	depends on maximum clock frequency number of cores, vectorization capabilities

## Data Availability

The original data presented in the study are openly available in UAV-TIRVis at https://gitlab.cs.pub.ro/etti/dcae-public/arh/research/uav-tirvis.git (accessed on 4 September 2025).
